# Glutamine deprivation plus BPTES alters etoposide- and cisplatin-induced apoptosis in triple negative breast cancer cells

**DOI:** 10.18632/oncotarget.10579

**Published:** 2016-07-13

**Authors:** Lian Chen, Hengmin Cui, Jing Fang, Huidan Deng, Ping Kuang, Hongrui Guo, Xun Wang, Ling Zhao

**Affiliations:** ^1^ College of Veterinary Medicine, Sichuan Agricultural University, Ya'an 625014, China; ^2^ Key Laboratory of Animal Diseases and Environmental Hazards of Sichuan Province, Ya'an 625014, China

**Keywords:** glutamine, apoptosis, BPTES, cisplatin, etoposide

## Abstract

Glutamine provides cancer cells with the energy required to synthesize macromolecules. Methods which block glutamine metabolism in treatment of breast cancer inhibit oncogenic transformation and tumor growth. We investigated whether inhibiting glutamine metabolism produces effects that are synergistic with those produced by drugs which damage DNA in triple-negative breast cancer cells. HCC1937 and BT-549 breast cancer cells were co-treated with either cisplatin or etoposide in combination with BPTES (a specific inhibitor of glutaminase 1) or exposure to a glutamine-free medium, and the cell proliferation and cell apoptosis were measured by flow cytometry, immunoblotting studies, and CCK-8 assays. The results showed that both glutamine deprivation and BPTES pretreatments increased the toxic effects of cisplatin and etoposide on HCC1937 cells, as demonstrated by their reduced proliferation, increased expression of apoptosis-related proteins (cleaved-PARP, cleaved-caspase 9, and cleaved-caspase 3) and decreased Bcl-2/BAX ratio. However, in BT-549 cells, glutamine deprivation and BPTES treatment increased etoposide-induced apoptosis only when used with higher concentrations of etoposide, and the effect on cisplatin-induced apoptosis was minimal. These results suggest that the anti-cancer effects produced by a combined approach of inhibiting glutamine metabolism and administering common chemotherapeutic agents correlate with the tumor cell type and specific drugs being administered.

## INTRODUCTION

Breast cancer is the second most common cancer worldwide, and women with this disease exhibit a high rate of disease relapse [1]. Oncologists classify breast cancer tissue based on its levels of estrogen receptors, progesterone receptors, and human epidermal growth factor receptor-2 expressions in biopsy specimens, and use that information to classify breast cancers into categories of triple-negative breast cancer, luminal breast cancer, and HER2^+^ breast cancer [2]. Triple-negative breast cancer accounts for approximately 20% of breast cancer cases [3] and comprises claudin-low and basal-like subtypes [3]. Triple-negative breast cancer is the most aggressive type of the disease and the only class treated with chemotherapy alone [2]. Moreover, there is currently no specific targeted therapy for triple-negative breast cancer, and the infected patients have a poor prognosis [4].

Glutamine is a non-essential amino acid which serves as a precursor for the synthesis of many amino acids, proteins, and nucleotides. It also participates in gluconeogenesis and helps to provide oxidative fuel (NADPH and NADH) for rapidly proliferating cells and tissues, as well as for glutathione synthesis [5]. Most cancers, including the most aggressive forms of breast cancer, require a constant supply of glutamine to support cell growth and proliferation. Increasing evidence suggests that specific alterations of glutamine metabolism in cancer cells provide potential methods for treating cancers. As a result, the inhibition of glutamine metabolism has become a “hot area” of cancer research. Various inhibitors of glutaminase and glutamate dehydrogenase enzymes, as well as glutamine transporters, have been proven effective for inhibiting the growth of cancer cells [6–8]. Glutaminase inhibitors, including DON, compound 968 and BPTES have been reported [9–11], and a new inhibitor (CB-839) has been studied by Gross et al. [6]. DON is nonspecific and inhibits several enzymes that utilize glutamine [12], while compound 968 is a specific inhibitor of glutaminase c (a subtype of glutaminase 1) [13]. BPTES inhibits both types of glutaminase 1, including KGA and GAC [14]. Cells treated with BPTES show repressed glutamine uptake [15], reduced GSH levels, and elevated levels of reactive oxygen species [16]. Several studies have shown that BPTES can significantly inhibit the growth of xenograft tumors initiated with c-Myc-transformed lymphoma cells [16], and induce apoptosis in IMR90-ERMYC and HA1E-MYCER cells in a MYC-dependent manner. Furthermore, cells without MYC display lower rates of apoptosis [17], which indicates the important role played by MYC in BPTES-induced cell death. Glutamine deprivation has also been proven to induce cell death or cause synergistic effects in various types of cancer cells when used in combination with chemotherapeutic drugs [18–25].

Cisplatin and etoposide are two drugs which damage DNA and are widely used in cancer therapy; however, increasing resistance to the effects of these drugs over time limits their use. To increase the sensitivity of cancer cells to these agents, cisplatin and etoposide are often co-administered to patients or given in combination with other drugs. In this study, we investigated the sensitivities of a claudin-low breast cancer cell line (BT-549) and a basal-type breast cancer cell line (HCC1937) to cisplatin and etoposide when administered under conditions of glutamine deprivation. We also examined the effects of BPTES on etoposide- or cisplatin-induced apoptosis in HCC1937 and BT-549 breast cancer cells. Our results showed that glutamine deprivation or BPTES pretreatment increased the sensitivity of HCC1937 cells to sub-toxic doses of cisplatin and etoposide, but had limited effects on the sensitivity of BT-549 cells.

## RESULTS

### Etoposide and cisplatin inhibited the proliferation of breast cancer cells

**Figure 1:**
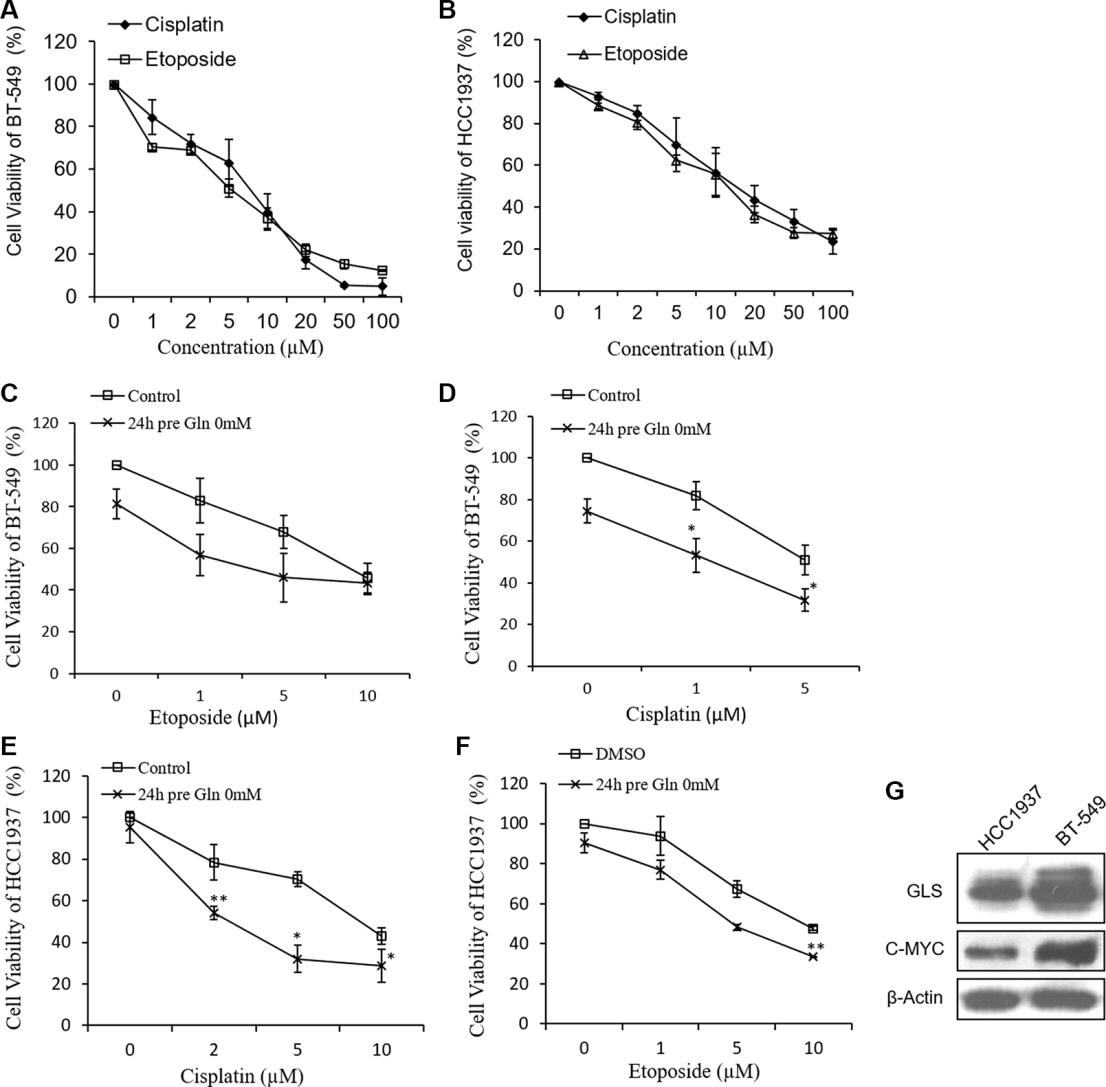
Growth inhibitive curve of cisplatin, etoposide and glutamine deprivation against HCC1937 and BT-549 cells Cell viability of HCC1937 cells (**A**) and BT-549 cells (**B**) are measured by CCK-8 after cisplatin or etoposide treatment for 48 hours. Glutamine free medium pretreatment for 24 hours increases cisplatin- and etoposide-induced cell proliferation inhibition in BT-549 cells (**C**, **D**) and HCC1937 cells (**E**, **F**). (**G**) Expressions of glutaminase (GLS) and c-MYC in HCC1937 and BT-549 cells. Data are expressed as means ± S.D. β-Actin is used as loading control. *p < 0.05, **p < 0.01 compare to control or DMSO.

We first investigated the effects of etoposide and cisplatin on breast cancer cell proliferation. HCC1937 and BT-549 cells were treated with various concentrations of etoposide or cisplatin for 48 h, after which their viability was measured by use of the CCK-8 assay. Our results showed that both compounds inhibited cell proliferation in a concentration-dependent manner. The IC_50_ values of cisplatin and etoposide when incubated with HCC1937 cells for 48 h were 14.77 ± 1.12 μM and 11.16 ± 1.19 μM, respectively, and when incubated with BT-549 cells were 6.04 ± 1.05 μM and 7.49 ± 1.08 μM, respectively (Figure 1A and 1B).

### Glutamine deprivation increased the abilities of cisplatin and etoposide to inhibit breast cancer cell proliferation

As triple negative breast cancer cells exhibit greater dependence on glutamine than other types of breast cancer cells [6], we examined the effects of glutamine deprivation on the abilities of cisplatin and etoposide to inhibit cell proliferation. In the initial studies, HCC1937 and BT-549 cells were pretreated with glutamine-free medium for 24 h, and then treated with different concentrations of cisplatin or etoposide for 48 h, after which cell proliferation was measured. As shown in Figure 1C–1F, HCC1937 cell proliferation was only slightly inhibited by glutamine deprivation, whereas BT-549 cell proliferation was more strongly inhibited. HCC1937 and BT-549 cells cultured in glutamine-free medium for 24 h displayed greater inhibition of cisplatin- and etoposide-induced cell proliferation than did cells that had not been cultured in glutamine-free medium, suggesting the synergistic effects of these treatments.

### Glutamine deprivation altered etoposide- and cisplatin-induced apoptosis in BT-549 and HCC1937 cells

**Figure 2:**
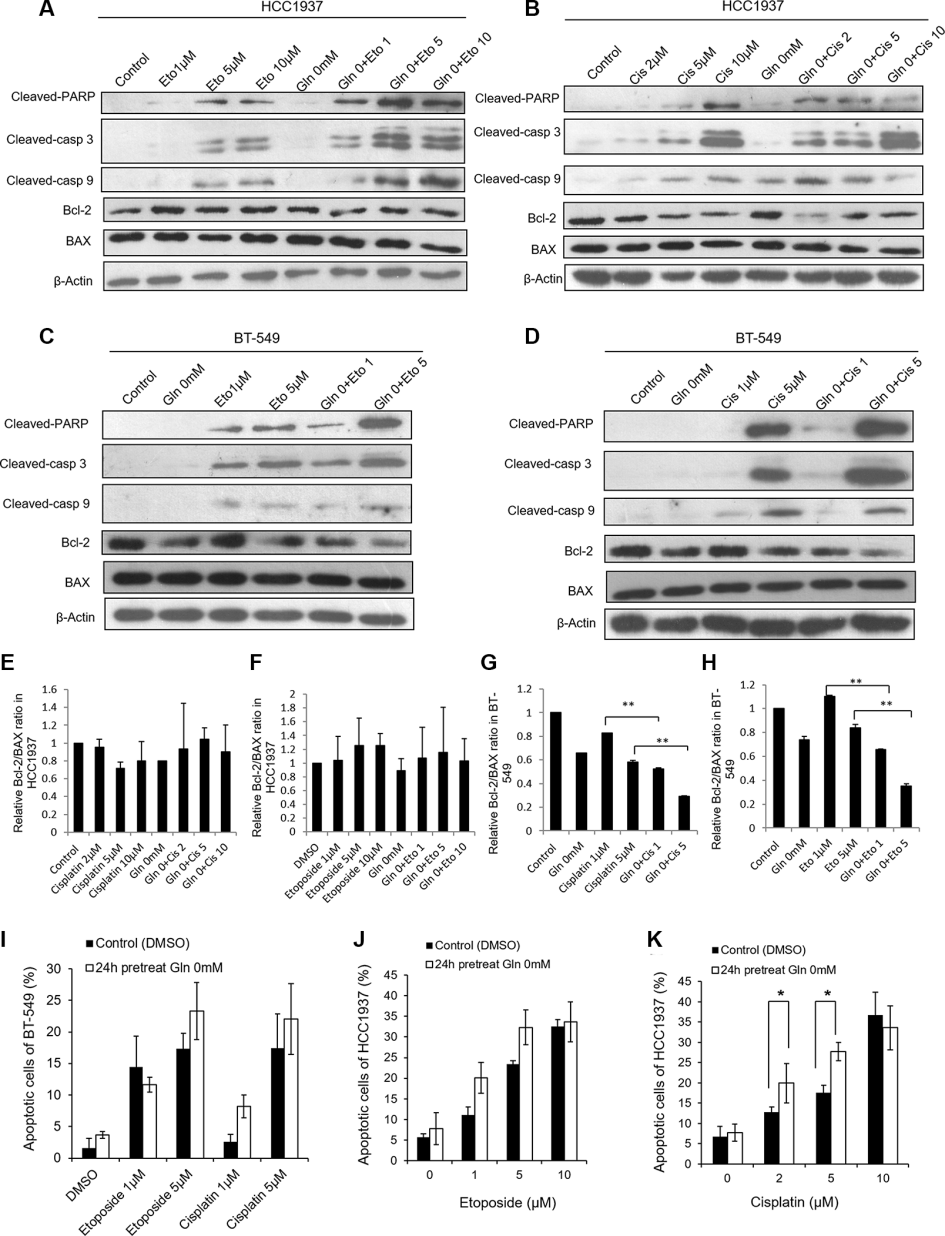
Glutamine deprivation alters apoptosis reactions in HCC1937 and BT-549 cells caused by cisplatin or etoposide HCC1937 and BT-549 cells are cultured in glutamine free medium for 24 hours, and then treated with cisplatin or etoposide for 48 hours. Representative blots show the expressions of cleaved-PARP, cleaved-caspase 3, cleaved-caspase 9, BAX and Bcl-2 in HCC1937 cells (**A**, **B**) and BT-549 cells (**C**, **D**) under glutamine deprivation condition. Relative Bcl-2/BAX ratio measured by immunoblotting in HCC1937 cells (**E**, **F**) and BT-549 cells (**G**, **H**). Cell apoptosis are measured by flow cytometry in BT-549 cells (**I**) and HCC1937 cells (**J**, **K**). Data are expressed as means ± S.D. Cleaved-casp 9, cleaved-caspase 9; cleaved-casp 3, cleaved-caspase 3. β-Actin is used as loading control. *p < 0.05, **p < 0.01.

To determine the mechanism by which glutamine deprivation altered etoposide- and cisplatin-induced cell proliferation, we examined whether glutamine deprivation could increase the levels of etoposide- and cisplatin-induced cell apoptosis. Based on their IC_50_ values, cisplatin and etoposide were each tested at concentrations of 1 μM and 5 μM with BT-549 cells, and at 2 μM, 5 μM, 10 μM (Cisplatin) and 1 μM, 5 μM, 10 μM (Etoposide) concentrations with HCC1937 cells. As shown in Figure 2A–2D, glutamine deprivation by itself induced a weak expression of apoptosis-related proteins in HCC1937 cells but not in BT-549 cells. Etoposide and cisplatin at the indicated concentrations each induced a moderate degree of apoptosis in HCC1937 cells (Figure 2A and 2B). However, when glutamine was removed from the medium for 24 h, the expression levels of cleaved-PARP, cleaved-caspase 3, and cleaved-caspase 9 induced by etoposide at 1 μM, 5 μM, and 10 μM concentrations, and by cisplatin at 2 μM and 5 μM concentrations increased, while the expression levels of BAX and Bcl-2 did not change (Figure 2E and 2F). In contrast, the Bcl-2/BAX ratio in BT-549 cells was decreased under conditions of glutamine deprivation (Figure 2G and 2H), which indicated an ongoing apoptotic process. Additionally, BT-549 cells deprived of glutamine displayed slightly increased levels of etoposide-induced apoptotic protein expression at the higher concentration of etoposide (5 μM), as well as cisplatin-induced expression of apoptotic proteins (Figure 2C and 2D).

To further examine the apoptotic effects induced by glutamine deprivation when used in conjunction with etoposide or cisplatin treatment in HCC1937 and BT-549 cells, we detected apoptotic cells by use of Annexin V-PE/7-ADD or PI/Annexin V staining and flow cytometric methods. As shown in Figure 2I–2K, the observed effects were consistent with changes in protein expression. HCC1937 cells incubated with etoposide or cisplatin in glutamine-free medium displayed higher levels of apoptosis than did cells incubated with either drug in medium containing glutamine. The exception was cells incubated with 10 μM cisplatin, in which cases use of a glutamine-free medium did not further enhance apoptosis (Figure 2J and 2K). BT-549 cells incubated with cisplatin or 5 μM etoposide in glutamine-free medium displayed increased levels of apoptosis, but with no statistic difference compared with cisplatin or etoposide treatment in medium containing glutamine (Figure 2I). These results indicated that the effects of glutamine deprivation on etoposide- or cisplatin-induced apoptosis correlate with drug concentrations.

**Figure 3:**
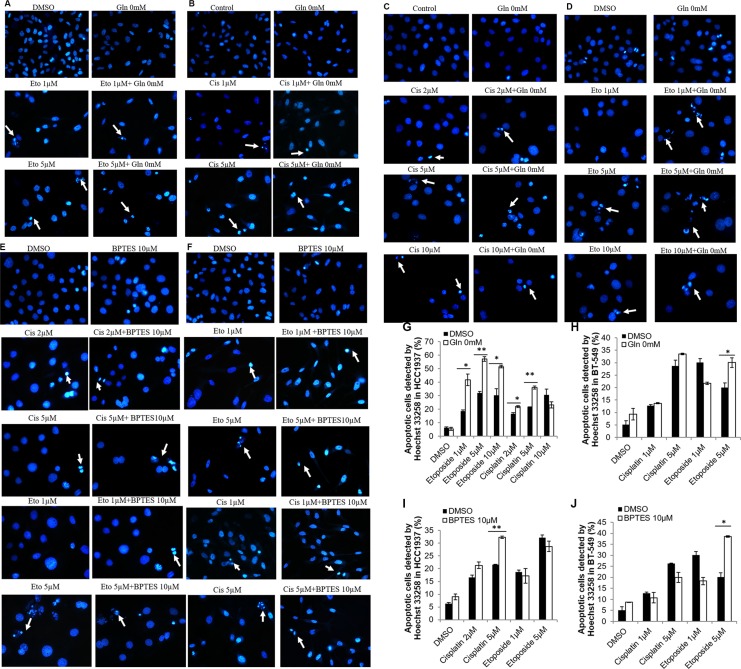
Morphological changes of HCC1937 and BT-549 detected by Hoechst 33258 Representative photomicrographs show cell shrinkage, condensation and fragmentation of the nuclei as well as the apoptotic bodies. Etoposide or Cisplatin, glutamine free medium and their combination produce apoptosis morphology in BT-549 cells (**A**, **B**) or HCC1937 cells (**C**, **D**). Cisplatin, etoposide, BPTES and their combination produce apoptosis morphology in HCC1937 cells (**E**) or BT-549 cells (**F**). Apoptotic cells were calculated in HCC1937 cells (**G**, **I**) and BT-549 cells (**H**, **J**). *p < 0.05, **p < 0.01. (×400).

Next, we used Hoechst 33258 staining methods to examine the morphological changes caused by simultaneous glutamine deprivation and cisplatin or etoposide treatment. Our results showed that while glutamine deprivation alone did not alter the morphology of HCC1937 and BT-549 cell nuclei, treatment of the cells with etoposide or cisplatin in a glutamine-free medium induced typical apoptotic changes, including cellular shrinkage, condensation, fragmentation of the nuclei, and the formation of apoptotic bodies (Figure 3A–3D and Figure 3G–3H).

Collectively, these results indicate that although glutamine deprivation only modestly altered the cell proliferation and apoptotic processes in HCC1937 and BT-549 cells, it sensitized HCC1937 cells to etoposide (1 μM, 5 μM, and 10 μM) and cisplatin (2 μM, 5 μM), and also increased the toxic effects produced by cisplatin and etoposide (5 μM) in BT-549 cells.

### BPTES combined with etoposide and cisplatin altered apoptosis in HCC1937 and BT-549 cells

**Figure 4:**
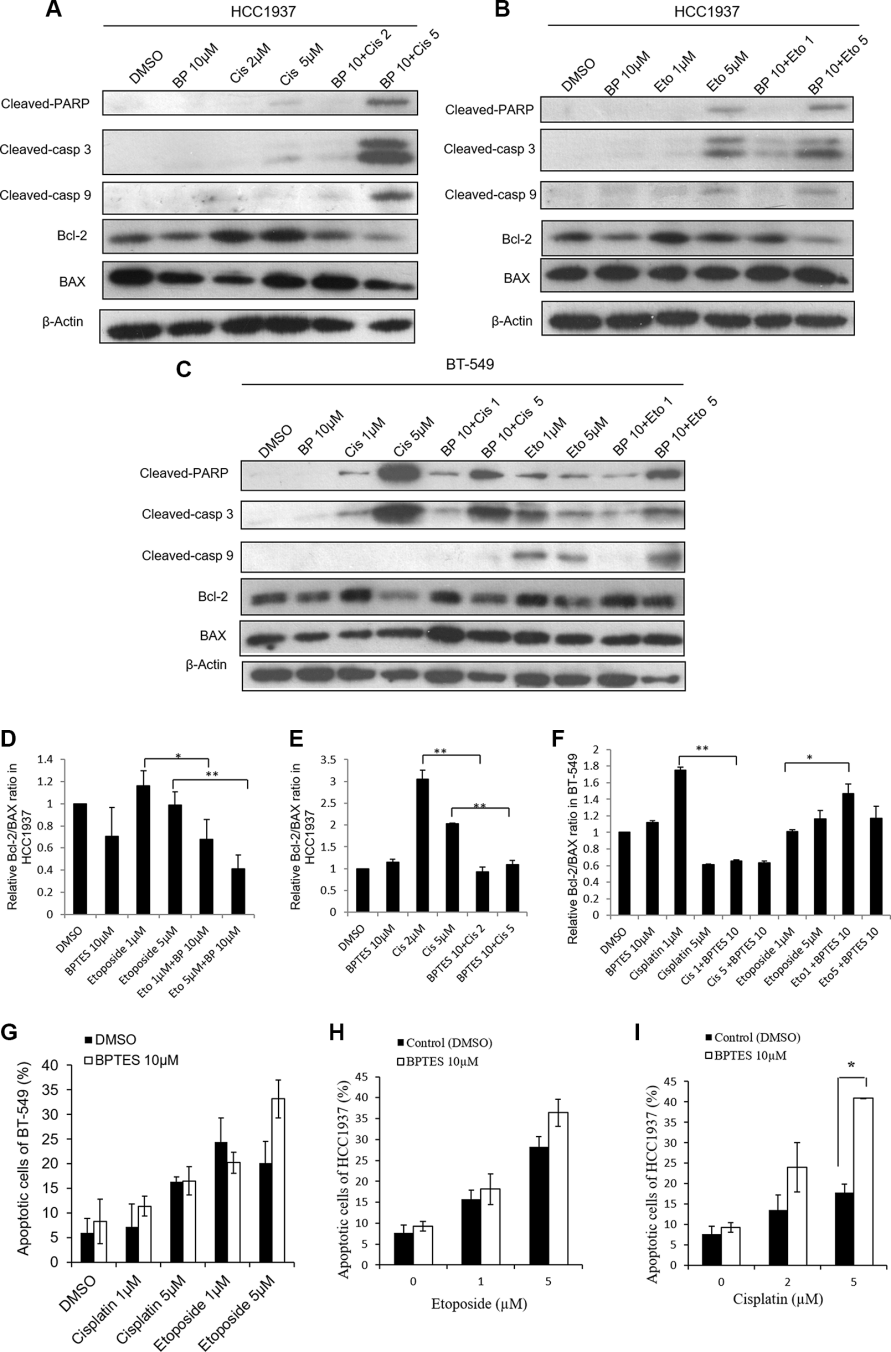
BPTES pretreatment alters apoptosis in HCC1937 and BT-549 cells caused by cisplatin or etoposide treatment for 48 hours HCC1937 and BT-549 cells are treated with 10 μM BPTES for 6 hours, and then subjected to cisplatin or etoposide for 48 hours. Representative blots show the expressions of cleaved-PARP, cleaved-caspase 3, cleaved-caspase 9, Bcl-2 and BAX in HCC1937 cells (**A**, **B**) and BT-549 cells (**C**). Relative Bcl-2/BAX ratio measured by immunoblotting in HCC1937 cells (**D**, **E**) and BT-549 cells (**F**). Cell apoptosis are measured by flow cytometry in BT-549 cells (**G**) and HCC1937 cells (**H**, **I**). Data are expressed as means ± S.D. Cleaved-casp 9, cleaved-caspase 9; cleaved-casp 3, cleaved-caspase 3. β-Actin is used as loading control. *p < 0.05, **p < 0.01.

To examine whether the glutaminase 1 inhibitor BPTES would have the same effects on etoposide- and cisplatin-induced apoptosis on cells cultured in glutamine-free medium, HCC1937 and BT-549 cells were pretreated with 10 μM BPTES for 6 h, and then with etoposide or cisplatin for 48 h. Because 5 μM and 10 μM concentrations of etoposide produced similar effects in HCC1937 cells, we selected the lower concentration (5 μM) in this experiment. As shown in Figure 4, pretreatment with BPTES sensitized HCC1937 cells to cisplatin (5 μM) and BT-549 cells to etoposide (5 μM), and also slightly increased the level of etoposide-induced apoptosis in HCC1937 cells, as evidenced by increased levels of cleaved-PARP, cleaved-caspase 3, and cleaved-caspase 9 expression, as well as a reduced Bcl-2/BAX ratio (Figure 4A–4F) and an increased level of apoptosis (Figure 4G–4I). However, BPTES did not alter the effects of cisplatin in BT-549 cells. Interestingly, pretreatment with BPTES reduced the level of apoptosis induced by a lower concentration of etoposide (1 μM) in BT-549 cells (Figure 4C). Moreover, treatment with BPTES alone resulted in weakened expression of apoptosis-related morphological changes, while BT-549 or HCC1937 cells treated with either etoposide or cisplatin in combination with BPTES decreased cell density and showed typical apoptosis cells (Figure 3E–3F and Figure 3I–3J). These findings indicated that, consistent with results of our glutamine deprivation studies, BPTES could increase the levels of etoposide- and cisplatin-induced apoptosis in breast cancer cells, in a manner dependent on the cell type being treated and the concentration of drugs administered.

## DISCUSSION

In the present study, we first determined the effects of glutamine deprivation on the viability of HCC1937 and BT-549 breast cancer cells treated with cisplatin or etoposide. We found that glutamine deprivation or treatment with BPTES plus cisplatin or etoposide resulted in increased cell death as demonstrated by CCK-8 staining and the expression levels of apoptosis-related proteins. Several studies have proven that interfering with glutamine metabolism can inhibit the growth of various types of cancer cells, including breast cancer [6, 9], non-small cell lung cancer [26], lung cancer [27], prostate cancer [7] and lymphoblastic leukemia cells [28, 29]. Due to its anaplerotic role in the TCA cycle, glutamine replenishes the intermediates needed by most cancer cells to synthesize macromolecules [30]. Therefore, reduced glutamine metabolism may limit the proliferation of cancer cells and thereby serve as a metabolic checkpoint that becomes activated in response to genotoxic stress [15]. Additionally, glutamine is metabolized to produce NADPH and GSH, which are needed to maintain oxidative homeostasis within a cell. Thus glutamine deprivation is sufficient to reduce GSH levels [31] and may result in oxidative stress and sensitize cells to etoposide and cisplatin.

Etoposide and cisplatin are both DNA damage drugs, which can induce DNA-double strand breaks. Cleaved-PARP has been shown the sole enzyme that can be immediately stimulated by DNA strand breaks [32]. In our study, cleaved-PARP expressions were detected in cells treated with etoposide and cisplatin, which were increased under glutamine deprivation or BPTES pretreatment condition. In accordance with cleaved-PARP expressions, cleaved-caspase3 was also increased, which is reported to promote PARP cleavage [33]. And as one of the apoptosis initiator, caspase-9 expression was also activated in our study. These results suggest involvement of intrinsic apoptotic pathways in present experiment. Glutamine deprivation can induce apoptosis in cancer cells via several mechanisms [18, 34–36], and the apoptosis pathways induced by glutamine deprivation are cell-specific. Glutamine starvation can cause BAX translocation into mitochondria, cytochrome c release, and increased caspase 9 and caspase 3 enzyme activity in Sp2/0 murine hybridoma cells [25]. However, in liver cancer cell lines, glutamine deprivation did not induce caspase-9 or -8 activation, but rather stimulated caspase-2 activity [22]. Similarly, our data showed that Bcl-2 and BAX may not contribute to the effects of glutamine deprivation plus etoposide or cisplatin in HCC1937 cells, but the Bcl-2/BAX ratio in HCC1937 cells was decreased when the cells were incubated with BPTES plus etoposide or cisplatin. Moreover, Bcl-2 and BAX were involved in BT-549 cell apoptosis. The Bcl-2/BAX ratio is believed to be more important than the expression levels of individual proteins and higher ratio of Bcl-2/BAX is skewed toward promoting cell survival [37]. Furthermore, elevated Bcl-2/BAX ratio appear to correlate with increased chemoresistance [38]. Therefore, in our study, a reduced Bcl-2/BAX ratio in conjunction with higher levels of caspase-3, caspase-9, and cleaved-PARP expressions indicated that an ongoing intrinsic apoptotic process.

Glutaminase is the first enzyme that participates in glutamine metabolism, and it has been proposed as a biomarker for glutamine-dependence, as well as a therapeutic target [4]. Interestingly, we found that either BPTES treatment or glutamine deprivation used in combination with sub-toxic doses of etoposide or cisplatin increased apoptosis in HCC1937 cells. This is consistent with a study which reported knock-down of glutaminase 1 with the use of small interfering RNA re-sensitized taxol-resistant breast cancer cells to taxol [39]. However, we found that pretreatment with BPTES had relatively limited effects on the toxic potencies of cisplatin and etoposide in BT-549 cells. It is known that BPTES specifically inhibits glutaminase 1, which suggests that glutaminase 2 is not suppressed. Furthermore, BPTES has been reported to increase the concentration of glycolytic intermediates in D54 cells and transformed normal human astrocytes [40]. Therefore, due to glycolysis compensation, BPTES may occasionally lose its ability to inhibit cell growth.

The degree of glutamine dependence exhibited by cancer cells is specific to the cell type, and glutamine restriction induces distinct reactions in different subtypes of breast tumors [4]. Therefore, it is not surprising that glutamine deprivation and BPTES pretreatments altered the effects of etoposide and cisplatin in HCC1937 cells to a greater extent than in BT-549 cells. Moreover, BPTES is a specific inhibitor of glutaminase 1, but not glutaminase 2. When compared with HCC1937 cells, BT-549 cells showed higher levels of glutaminase expression (Figure 1G), suggesting that higher concentrations of a glutaminase inhibitor are required to produce an anti-proliferative effect. Additionally, the sensitivity of tumors to chemotherapy, especially when administered at low doses, is largely dependent on their genotype or levels of oncogene and tumor suppressor gene expression [41]. Thus, the limited effects of glutamine deprivation and BPTES pretreatment on cisplatin or etoposide (1 μM) in BT-549 cells might be partially attributable to a high level of c-MYC expression (Figure 1G). The c-MYC oncogene (a transcription factor) participates in glutamine metabolism by increasing the expression levels of glutaminase 1 and the glutamine transporter (ASCT2) [42, 43]. Cisplatin-resistant tumor cells, e.g., NIH3T3 cells and metastatic melanoma cells, express high levels of the c-MYC protein [44–46], an observation which suggests that c-MYC overexpression contributes to chemoresistance.

In conclusion, our study demonstrates that a combination of glutamine deprivation and BPTES treatment sensitize HCC1937 cells to sub-toxic doses of etoposide and cisplatin by upregulating the intrinsic cellular apoptosis pathway. Furthermore, BT-549 cells display concentration- and drug-dependent responses to inhibition of glutamine metabolism. These data strongly suggest that an inhibitor of glutamine metabolism could be used in conjunction with standard chemotherapy as a strategy for treating triple-negative breast cancer; however, the effects of cell specificity and drug concentration must be taken into account.

## MATERIALS AND METHODS

### Reagents and antibodies

Cisplatin, BPTES and DMSO were purchased from Sigma-Aldrich (St. Louis, MO, USA). Etoposide was purchased from Aladdin (Shanghai, China). Antibodies against cleaved-caspase 3 (9664), cleaved-PARP (5625), BAX (2772), Bcl-2 (2870), β-actin (4970), cleaved-caspase 9 (7237) and peroxidase-conjugated secondary antibodies were purchased from CST (Cell Signaling Technology). Antibodies against c-MYC (ab32072) and GLS (ab156876) were purchased from Santa Cruz Biotechnology (Santa Cruz, CA, USA). A stock solution of BPTES (25 μM) was prepared by dissolving BPTES in DMSO. Cisplatin were prepared by dissolving cisplatin in 0.9% sodium chloride.

### Cell lines and culture

BT-549 and HCC1937 were purchased from Shanghai Cell Collection (Shanghai, China). Cells were cultured in RPMI 1640 medium (11875-093, Gibco, USA) supplemented with 10% of FBS (Gibco, USA) and 100 U/mL penicillin, and 100 μg/mL streptomycin. For BT-549 cells, 0.023IU/ml insulin was additionally added to the medium. For glutamine deprivation, cells were cultured in RPMI 1640 medium without glutamine (21870-076, Gibco, USA) supplemented with 10% of FBS (Gibco, USA) and 100 U/mL penicillin, and 100 μg/mL streptomycin. All cells were maintained in a humidified incubator at 37°C with 5% CO_2_ and passaged with 0.25% trypsin-EDTA.

### Cell viability assay

Cell viability were measured by CCK-8 assay as described previously [47, 48]. Aliquots containing 5 × 10^3^ cells in 100 μL of medium were seeded into 96-well cell culture plates, and the next day were treated for 48 h with the indicated concentrations of etoposide or cisplatin. When the effects of glutamine deprivation were studied, 5 × 10^3^ cells in 100 μL of medium were seeded into 96-well cell culture plates, after which they were transferred to glutamine-free medium the next day and cultured for an additional 24 h. After culture, the cells were treated with various concentrations of etoposide or cisplatin for 48 h, after which they were incubated with 10 μL of CCK-8 agents (Beyotime; Jiangsu, China) in 100 μL of medium for 1 h at 37°C. After incubation, the absorbance of each sample was measured at a wavelength of 450 nm with a Thermo Fisher ScientificMultiskan™ microplate spectrophotometer (Thermo Fisher Scientific; Waltham, MA, USA). The experiment was performed in triplicate.

### Apoptosis assay

The percentage of apoptosis was evaluated by using an Annexin V-PE/7-AAD or Annexin V-FITC/PI staining detection Kit (BD Biosciences, San Jose, CA, USA) according to the manufacturer's protocol. Cells were treated with agents for indicated time, and then harvested and washed two times with PBS. Pellets were collected and resuspend in PBS, and then stained with PE Annexin V and 7-AAD or Annexin V and PI for 15 minutes in the dark. Data were then obtained by a FACSCalibur (Becton Dickinson, USA).

### Immunoblotting

Total soluble proteins were extracted from harvested cells and stored in Laemmli sample buffer. Protein samples used for analysis were heated for 5 min at 99°C, after which equal quantities of protein as determined using the bicinchoninic acid assay were separated by 10%–15% SDS-PAGE. The separated protein bands were transferred onto nitrocellulose filter membranes, which were then blocked with 5% fat-free milk in PBS-Tween for 1 h. Next, the membranes were incubated with the relevant primary antibodies overnight, washed with PBS-Tween, and incubated with the corresponding HRP-linked secondary antibody for 1 h. After incubation, the membranes were washed with PBS-Tween, and the blots were visualized with ECL^TM^ (Bio-Rad; Hercules, CA, USA) and X-ray film.

### Hoechst 33258 staining

Cells were cultured on glass coverslips in a 6-well plate and treated with the indicated agents for various time periods. Following treatment, the cells were washed with PBS and fixed with 4% paraformaldehyde for 10 min. The fixed cells were then washed twice with PBS and stained with Hoechst 33258 solution (10 μg/mL, Beyotime; Jiangsu, China) for 10 min in the dark. The stained cells were washed with PBS, and their nuclear morphology was observed with an Olympus fluorescence microscope (Olympus; Tokyo, Japan).

### Statistical analysis

All data were presented as mean ± SD. All assays were performed for at least three times. Differences between control and experimental groups were determined by one-way analysis of variance (ANOVA). *p* < 0.05 was considered statistically significant, *p* < 0.01 was considered statistically highly significant.

## Abbreviations

7-ADD: 7-amino-actinomycin
968: 5-(3-bromo-4-(dimethylamino)phenyl)-2,2-dimethyl-2,3,5,6-tetrahydrobe nzo[a]phenanthridin-4(1H)-one
ASCT2: System ASC amino acid transporters 1 and 2
BAX: Bcl-2 associated X protein
Bcl-2: B-cell lymphoma-2
BPTES: Bis-2-(5-phenylacetamido-1,2,4-thiadiazol-2-yl)ethyl sulfide 3
CB-839: N-(5-(4-(6-((2-(3-(Trifluoromethoxy)phenyl)acetyl)amino)-3-pyridazinyl)butyl)-1,3,4-thiadiazol-2-yl)-2-pyrid ineacetamide)
DMSO: Dimethyl Sulphoxide
DON: 6-diazo-5-oxo-l-norleucine
EDTA: Ethylenediam inetetraacetic acid
FBS: Fetal bovine serum
GAC: Enlongated kidney glutaminase variant
GLS: Glutaminase
GSH: Glutathione
HER2: Human epidermalgrowth factor receptor-2
HRP: Horseradish Peroxidase
IC50: Half maximal inhibitory concentration
KGA: Kidney glutaminase
NADH: Nicotinamide-adenine dinucleotide
NADPH: Nicotinamide adenine dinucleotide phosphate
NSCLC: Non-small-cell lung cancer
PARP: Poly ADP-ribose polymerase
PBS: Phosphate buffered saline
SDS-PAGE: Sodium dodecyl sulfate polyacrylamide gel electropheresis
TCA: Tricarboxylic acid cycle
